# Differential Allocation by Female Zebrafish (*Danio rerio*) to Different-Sized Males – An Example in a Fish Species Lacking Parental Care

**DOI:** 10.1371/journal.pone.0048317

**Published:** 2012-10-26

**Authors:** Silva Uusi-Heikkilä, Linda Böckenhoff, Christian Wolter, Robert Arlinghaus

**Affiliations:** 1 Department of Biology and Ecology of Fishes, Leibniz-Institute of Freshwater Ecology and Inland Fisheries, Berlin, Germany; 2 Unit of Evolutionary Biology/Systematic Zoology, Institute of Biochemistry and Biology, University of Potsdam, Potsdam, Germany; 3 Inland Fisheries Management Laboratory, Department for Crop and Animal Sciences, Faculty of Agriculture and Horticulture, Humboldt-Universität zu Berlin, Berlin, Germany; Tulane University Medical School, United States of America

## Abstract

Organisms allocate resources to reproduction in response to the costs and benefits of current and future reproductive opportunities. According to the differential allocation hypothesis, females allocate more resources to high-quality males. We tested whether a fish species lacking parental care (zebrafish, *Danio rerio*) expresses male size-dependent differential allocation in monogamous spawning trials. In addition, we tested whether reproductive allocation by females is affected by previous experience of different-quality males, potentially indicating plasticity in mate choice. To that end, females were conditioned to large, small or random-sized males (controls) for 14 days to manipulate females' expectations of the future mate quality. Females showed a clear preference for large males in terms of spawning probability and clutch size independent of the conditioning treatment. However, when females experienced variation in male size (random-sized conditioning treatment) they discriminated less against small males compared to females conditioned to large and small males. This might suggest that differential allocation and size-dependent sexual selection is of less relevance in nature than revealed in the present laboratory study.

## Introduction

Sexual selection represents selection for traits that increase an individual's reproductive success. In many mating systems, males compete with each other for access to females (i.e., intra-sexual selection), and femal--es in turn select the most attractive male to mate with (i.e., inter-sexual selection) [1]. Female mate choice is believed to have evolved because it provides females with direct, material benefits (e.g., nutrition, parental care) [2], [3] or indirect, genetic benefits (sexy son and good genes hypotheses) [4], [5], [6] that collectively should increase female's fitness. In response to sexual selection, males of many species develop a variety of secondary sexual characters and traits, ranging from body ornaments (e.g., kype and adipose fin in Atlantic salmon, *Salmo salar*) [7] to distinct coloration (e.g., in guppies, *Poecilia reticulata*) [8], to signal their attractiveness as mating partners. In species that lack obvious secondary sexual traits, male body size may constitute an important sexually selected character. Large males of some fish species have indeed been shown to provide females with direct fitness benefits, for example better nests (e.g., in minnow, *Pimephales promelas*) [9] or more intensive offspring care (e.g., in smallmouth bass, *Micropterus dolomieui*) [10]. In species where material benefits offered by males and male body size correlate positively, females are consequently expected to prefer mating with a large male (e.g., minnow) [9]. However, females may exercise size-dependent mate choice also in the absence of any obvious material resources offered by the male (i.e., species with a resource free mating system), in which case mating preferences must be a result of indirect, e.g. genetic, benefits offered by the male [5].

Reproduction is energetically costly and usually affects future growth and survival negatively [11]–[13]. Therefore, individuals should weigh costs and benefits of investing in reproduction with their current mate against the expected quality and corresponding fitness prospects offered by future mates [14]. According to the differential allocation (DA) hypothesis, females are expected to invest more when they are paired with high-quality males compared to low-quality males, thereby generating a positive relationship between partner quality and reproductive investment [14], [15]. A theoretical model has suggested that DA should be an optimal strategy under many environmental conditions unless future mate choice is strongly constrained [16]. In particular, if the likelihood for future mating opportunities or the expected future mate quality is very low, then females might invest more reproductive resources when mating with low-quality mates, known as reproductive compensation [17]. Reproductive compensation should, however, be rare in nature [16]. In the wild, an individual usually experiences variation in the attractiveness of potential mates it can breed with over its lifetime, but selection should nevertheless favor individuals that allocate resources towards mates offering greater fitness prospects [15]. Wide-range empirical support for DA has been obtained across many animal species [15], [18].

Considering the diversity of mating systems in fish, surprisingly few experimental studies have been conducted on the DA hypothesis in this taxon, and especially few studies are present in species which lack parental care or other obvious male-contributed spawning resources (but see [19], [20]). However, rainbow fish (*Melanotaenia australis*) males are not known to provide any obvious material resources to females but it has been shown that females spawn more eggs to large males compared to small males [20]. Similarly, zebrafish (*Danio rerio*) is a batch spawning fish with no parental care [21], and although the species lacks clear secondary sexual characteristics, zebrafish females have been shown to discriminate particular male traits during spawning [22], [23]. Some studies have shown females to prefer large males [24], whereas others have reported contradictory findings, both in terms of association preference [25] and spawning success [23]. However, in their pioneering study on DA in zebrafish, Skinner and Watt [19] showed that when zebrafish females were mated sequentially with a large and a small male, they released a greater number of eggs to the second male when he was large-sized, while no DA allocation was present in the first mating. Females who released more eggs during the second spawning trial in the study by Skinner & Watt [19] were the ones that were coupled with small males first. The DA revealed in the second trial could be related to a previous male effect, as evidenced by a significant mating group effect, or to the time of mating. Because DA was not expressed in the first spawning, the evidence for DA in zebrafish remains weak.

The DA-pattern can be expected to express certain degree of flexibility. Previous experience [26]–[28] or social learning patterns [29], [30], in particular, may cause experience-dependent plastic changes in female mate preferences over time. If females are forced to sample potential mates sequentially and to compare the present mate with the ones she met previously, she is likely to rate males based on the attractiveness of a previous male, thus exhibit some plasticity in mate choice [26], [31]. In sticklebacks (*Gasterosteus aculeatus*), female's internal standards for male quality have been found to be adjustable so that a given male is rated higher by a female when preceded by a less attractive than by an attractive male (previous male effect) [31]. Furthermore, mating preferences can be lost or reversed when the social environment changes. In particular, any social environment consisting of different-sized males may mediate the expected future mate quality and thus influence female's allocation pattern in species where male size signals high quality [28].

In the present study, we tested 1) whether females express DA, using zebrafish as an example of a species lacking parental care and other obvious male-derived mating resources and 2) whether the allocation pattern is plastic and can be altered by manipulating the expected future mate quality by conditioning females to various male sizes. We considered DA as a general strategy for many iteropareous organisms that face variation in mate quality. However, such preference might be plastic and amendable to change by manipulating the expected future mate quality (i.e. exposure to different male sizes), so that females held under constrained availability of high quality males may show plasticity in their allocation pattern and express less distinct DA pattern. Despite the fact that current resource allocation is predicted to depend on the expected future mate quality [15], the effects of female experience, which is likely to be determined by the past experience, has been somewhat overlooked in the DA studies (but see [28], [32]). Our study is among the few to focus on a fish species with a largely resource free mating system where DA is expected to be less pronounced than in mating systems where males of different qualities provide material benefits to females (e.g., nest guarding).

## Materials and Methods

### Ethics Statement

No permits were necessary for the experimental work as it did not harm or induce stress or suffering to the animals. No observation of fish was conducted in the field, this observational study was based solely on captive-bred fish. Fish were housed in conditions (see “Experimental Fish and Holding Conditions”) that comply with the current animal welfare laws, guidelines and policies of Germany.

### Experimental Fish and Holding Conditions

Our experimental fish were the third generation offspring from a wild zebrafish population captured from a river system 70 km west of Coochbihar (West-Bengal, India, 22.56°N, 87.67°E). Fish (females and males) were raised in glass fiber – polyester tanks (volume 320 l) in a light (14 h light : 10 h dark) and temperature controlled (mean±s.d. 26.8±0.79°C) recirculation facility with an inflow rate of 0.25 ls^−1^. The stocking density was 0.9±0.2 individuals l^−1^. Fish were fed *ad libitum* with Artemia nauplii (Inve Aquaculture NV) and commercial flake food (TetraMin, Tetra GmbH; 47% protein, 10% fat).

### Conditioning Females to Different-sized Males

To study whether manipulating the expected future mate quality changes female mating preferences or allocation pattern of reproductive resources, we conditioned females to social environments, which consisted of different-sized males (random-sized, large and small) for 14 days. These social environments are referred to as three different conditioning treatments, where in one treatment same-sized females were held together with large males, in one treatment females were held together with small males and in the control treatment females were held together with random-sized (i.e., large and small) males (Table 1). Males were selected for the treatments based on their body length and each male was measured to the nearest mm. During the conditioning period, the fish likely spawned with each other, thus females had previous spawning experience. The conditioning time of 14 days was chosen because zebrafish can learn tasks in as few as 10 days and display rapid, reliable food conditioning [33], [34] and alarm reactions [35]. We thus assumed that zebrafish females would also be able to internalize social preferences and male size structure in a conditioning period of 14 days.

**Table 1:** The standard length (SL, mean±sd and range) of females and males used in the three different conditioning treatments.

Conditioning treatment	Females SL	Males SL
**Random-sized males**		
Mean±sd	27.2±0.69 mm	25.0±2.49 mm
Range (min–max)	27.0–30.0 mm	22.0–29.0 mm
	N = 17	
**Large males**		
Mean±sd	28.2±1.45 mm	27.1±1.54 mm
Range (min–max)	27.0–32.0 mm	26.0–34.0 mm
	N = 20	
**Small males**		
Mean±sd	27.6±1.17 mm	22.7±1.31 mm
Range (min–max)	27.0–31.0 mm	20.0–24.0 mm
	N = 20	

N indicates the number of individual couples used in the spawning trials.

It has been suggested in theoretical models that female and male age may mediate mate preference [36] and allocation patterns [16], [37]. Therefore, the fish used in our experiment were all the same age (150 days post fertilization; dpf). Female zebrafish start maturing at age 90 dpf [38] and at length of 19 mm [39]. Therefore, we were confident that all the females used in our experiment were mature (standard length mean±s.d.: 27.7±1.21 mm). Owing to the potentially aggressive, female stress-inducing behavior exhibited by the relatively largest and thus most dominant males in zebrafish [40], the body size of males assigned to the different conditioning treatments was controlled such that males were not larger than females (Table 1).

After the females and males were randomly assigned to the different conditioning treatments, the fish were stocked in aquaria (volume: 45 l), which were controlled for light (14 h light : 10 h dark) and temperature (mean±s.d. 25.6±1.13°C). The stocking density was 1.1 individuals l^−1^ with the 1∶1 sex ratio. In order to block the olfactory cues among the conditioning treatments, fish assigned to different treatments were held in separate recirculation systems (inflow rate of 0.14±0.43 l s^−1^), and additionally the aquaria were covered to prevent any visual contact among fish during the conditioning.

### Spawning Period and Data Collection

After 14 days of conditioning the females to different-sized males, a two day spawning trial was initiated to study the potential differences in reproductive allocation among females originating from different social environments (i.e., conditioning treatments). To obtain a maximal reproductive output in such a short time period, females were isolated from males for 24 hours [41] after which the fish were transferred into spawning boxes (volume: 3 l) designed to prevent egg cannibalism by separating the spawning fish from the eggs by a mesh construction (Aquarien-Bau Schwarz, 37081 Göttingen, Germany). Individual females from different conditioning treatments (i.e., held with random-sized, large or small males) were stocked into the spawning boxes either with a large male (27.2±1.41 mm) or a small male (22.6±1.06 mm). The difference in body length between large and small males was statistically significant (t_105.9_ = 20.81, *p*<0.001). It has been shown that familiarity can influence fish behavior and breeding performance [42], [43], therefore the males used in the spawning trial were novel individuals (i.e., held separately) to avoid any bias in reproductive performance caused by potential mate familiarity. The males used in the spawning trials were held together in separate systems without females, thus they had not spawned during the past 14 days. Large (t_87.07_ = −0.303, *p* = 0.762) and small males (t_122.7_ = 1.337, *p* = 0.184) used for conditioning and for spawning did not differ in their standard length. From the conditioning treatment where females were held with large males, 20 females were selected and half of them were coupled with a large male and the other half were coupled with a small male for the spawning trial (Table 1). The same number of females (20) was used from the conditioning treatment where females were coupled with small males (Table 1). From the random-sized male conditioning treatment, ten females were coupled with a small male and seven females were coupled with a large male (Table 1). Spawning boxes were stocked with one female and one male. Visual and chemical contact was prevented among the boxes during the two days spawning period.

Zebrafish spawn within the first few hours after sunrise [44], thus the assessment of reproductive output took place between 0800 and 1000 hours (automatic light-on in the spawning facility was at 0600 hours). The spawning boxes were cleaned on both days, the occurrence of spawning was assessed and the number of eggs was counted. To assess the egg fertilization probability, we enumerated fertilized eggs from unfertilized eggs immediately after the eggs were collected.

### Statistical Analyses

We used generalized linear model (GLM) to determine the effect of conditioning treatment and male body size on spawning probability, clutch size (i.e., number of eggs per female per day) and egg fertilization probability. In all of the analyses, conditioning treatment (random-sized, large or small males), male size (large or small male) and their interaction were treated as fixed effects. Spawning probability, clutch size and egg fertilization probability was estimated for the two spawning days, thus the individual couple and spawning day were set as random effects to estimate the variation among couples and between the days that could not be related to the conditioning treatment or male body size. The amount of variance associated with the random variables was estimated through variance components. Couples which did not produce any eggs during the two-days spawning period were excluded from the clutch-size analysis. Clutch size was modeled through Poisson regression and spawning probability and egg fertilization probability were modeled through binomial regressions. If data were over-dispersed, the quasi-Poisson or quasi-binomial distributions were used. Statistical significance of fixed effects was determined by chi square test comparisons of successively simpler models, which agreed with Akaike Information Criterion (AIC) model selection methods.

In the results, mean values are presented with standard errors. All data were considered statistically significant at *p*<0.05. All statistical analyses were performed with R 2.13.1. with the lme4 package [45].

## Results

**Figure 1:**
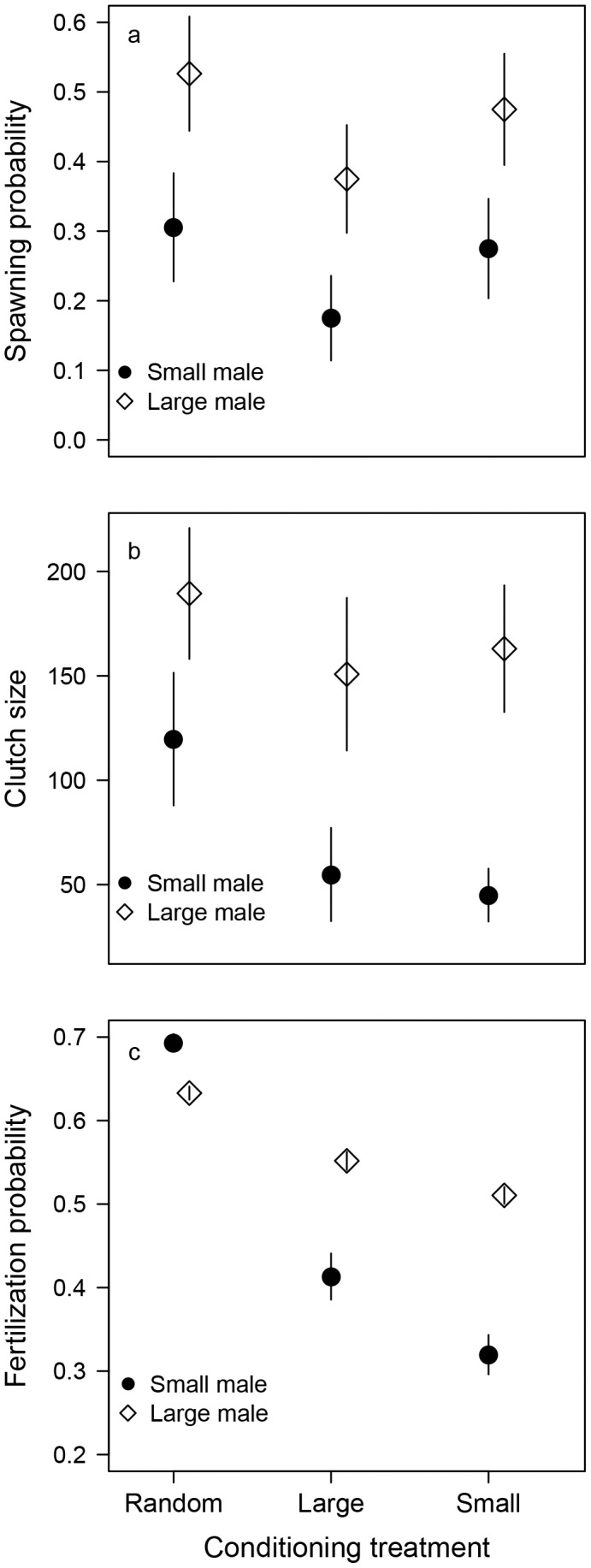
Female differential allocation pattern. a) average spawning probability, b) average clutch size per day, and c) average egg fertilization probability among females from different conditioning treatments coupled with either large or small males. Error bars indicate standard errors.

Male size during spawning trials had a significant effect on spawning probability, while the conditioning treatment did not affect it, and there was no interaction between the conditioning treatment and male size (Table 2). Spawning probability was higher when spawning occurred with large (0.46±0.05) relative to small males (0.25±0.04; Figure 1a, Table 2). A substantial amount of variation (76.3%) was associated with the individual couples. Spawning day was excluded from the model since virtually no variation was associated with this variable (<0.001%).

**Table 2:** The effect of conditioning treatment, male size and their interaction on reproductive parameters in zebrafish.

Trait	Variable	Estimated parameter values (SE)	?^2^-value^a^ (df)	*P*-value^b^
Spawning probability	Treatment		3.052 (5,3)	0.217
	**Male size**		9.123 (3,2)	0.003
	Small (Intercept)	−1.737 (0.350)		
	Large	1.465 (0.467)		
	Treatment×Male size		0.011 (7,5)	0.994
Clutch size	Treatment		5.246 (7,5)	0.073
	**Male size**		11.34 (5,4)	0.001
	Small (Intercept)	3.633 (11.28)		
	Large	1.162 (8.985)		
	Treatment×Male size		1.640 (9,7)	0.440
Fertilization probability	Treatment		5.148 (5,3)	0.076
	Male size		0.410 (6,5)	0.522
	Treatment×Male size		3.311 (8,6)	0.191

Significant predictors are indicated in bold.

a χ^2^ -value from the deletion of the variable from the full model

b P-values derived from the χ^2^ –statistics

The average clutch size (number of eggs produced by a female per day) was significantly higher among females crossed with large males (169±25.1) compared to females crossed with small males (94.9±18.5; Figure 1b, Table 2) when pooled across the conditioning treatments. The conditioning treatment did not have a significant effect on clutch size, although it approached statistical significance (*P* = 0.073, Table 2). Indeed, it appeared that the DA pattern in the conditioning treatment where females were held with random-sized males was less pronounced than in the conditioning treatments where females were held with large or small males. The interaction between conditioning treatment and male size did not have a significant effect on egg production (Table 2). In terms of clutch sizes, 48.7% of the variation was associated with the individual couples and 8.08% with the spawning days.

Conditioning treatment did not have a significant effect on egg fertilization probability, but as in clutch size, there was a strong trend (*P* = 0.076) in terms of differences in egg fertilization probability between large and small males. The difference was smaller in the conditioning treatment where females were held with random-sized males (large male: 0.63±0.01, small male: 0.70±0.01) compared to the conditioning treatments where females were held with either large (large male: 0.55±0.01, small male: 0.42±0.03) or small males (large male: 0.51±0.01, small male 0.32±0.02; Figure 1c). Neither male size nor the interaction between the conditioning treatment and the male size were significant (Table 2). In terms of egg fertilization probability, 56.6% of the variance was associated with the couples and 1.78% with the spawning day.

## Discussion

In accordance with our study hypothesis, we demonstrated that zebrafish females differentially allocate reproductive resources in terms of egg numbers based on the size-dependent attractiveness of their mate, and they also showed a greater propensity to spawn with larger males. The pattern of differential allocation (DA) was implicated by females releasing a greater number of eggs more frequently to large males compared to smaller conspecific males. We found this result to be robust against the male-size dependent social environment females were previously exposed to, although the DA pattern seemed to be less pronounced in the control treatment where females were exposed to random-sized males prior to spawning. Based on our results, larger males are more attractive mating partners for zebrafish females.

Our study is one of the few studies demonstrating the existence of DA in a species with a resource free mating system (see also [19], [20], [46]), thereby complementing the more contrived finding of DA previously reported in zebrafish by Skinner and Watt [19]. In their study, a female was coupled first with a small male and immediately after that with a large male. In a second treatment, a female was coupled first with a large male and then with a small male. Irrespective of the male size, females released more eggs to the first male. Before and during the consecutive spawning trials, females were able to sense the presence of an alternative male. Possibly, the females released equal amount of eggs for a less preferred male (small) in the first spawning, as spawning with a more preferred male (large) was perceived as possible in the future. In our study, females were isolated visually and chemically from other study males during the spawning trials, and unlike Skinner and Watt [19], we were able to show a clear pattern of DA that was independent of the conditioning treatment. Accordingly, our results were not confounded by female's assessment of the next male. Although females conditioned to large males (28.2 mm) were on average slightly larger compared to females conditioned to small (27.6 mm) and random-sized males in our study (27.2 mm, Table 1), females conditioned to large males expressed a similar level of DA relative to females conditioned to small males (Figure 1).

We found that the DA by female zebrafish was less pronounced among females held with random-sized males where the conditioning treatment consisted of both small and large males. These females appeared to be less discriminative towards small males potentially because they were trained to expect the possibility to encounter a large male in the future by experiencing continuous variation in male size. This experience may have not promoted perceptual discrimination against unpreferred [24] small males. By contrast, females held with small males expressed a clear DA pattern indicated by their higher spawning probability and greater egg allocation when the quality of the male encountered was higher (large male) than the expected future mate quality (small male). Females conditioned to large males, on the other hand, may have developed a threshold criterion [47], [48] according to which they do not mate or at least allocate less reproductive resources to males not exceeding the threshold (small male). Obviously we can only speculate about the plausible explanations regarding the previous male effect [26] but the fact that females held with random-sized males seemed to be less discriminative in terms of allocating reproductive resources (e.g., clutch size) suggests some degree of socially mediated plasticity in zebrafish DA (see also [19]), which possibly reduces the prevalence of DA under more natural conditions.

Our study results are not likely methodological artifacts because the reproductive output revealed in the present experiment was comparable to reproductive behaviors previously studied in our laboratory. In fact, females held with random-sized males in the present experiment had similar spawning probabilities on average compared to similar-sized females coupled with large and small males in a previous study [40]. However, females in the present study produced higher number of eggs compared to the females in the previous study [40]. The differences in egg numbers between these two studies could be caused by differences in the experimental set-ups, age of spawners and even seasonality. The higher clutch sizes in the present study compared to the previous one [40] could indicate high female condition and therefore, our study results seem to be unaffected by stress or other conditions negatively influencing reproductive output.

In various species, females can enhance their fitness by choosing a mate who can provide, for example, a better nest site or more intensive care for the offspring [49], [50]. The mechanism for why female zebrafish prefer [24] and strategically allocate eggs to larger males is less clear, as this species lacks obvious male-derived spawning resources. However, it has been shown in laboratory studies that zebrafish male body size correlates with territoriality [51] and with dominance [52]. Dominant and territorial males might provide females better oviposition sites. However, wild zebrafish males reared in semi-natural conditions have been shown rarely to express territoriality but mostly pursue females actively before and during spawning [25]. Thus, it is not clear whether territoriality is a typical behavioral strategy among zebrafish males in nature and whether it could explain the observed DA pattern. In addition to direct benefits, females may also receive indirect, genetic benefits from large males. According to the good genes hypothesis, females gain an evolutionary advantage by mating with a high-quality male and passing those genes on to the offspring [4], [5]. In zebrafish, large male body size can be associated with high male quality as large individuals are more dominant in the social hierarchy and thus are able to govern feeding opportunities [53]–[55]. Indeed, large (but not very large) males have been previously reported to exhibit higher reproductive success compared to small males [40], [51]. However, it was not shown whether the mechanisms behind the high reproductive success of large males were direct paternal effects or indirect maternal effects related to female DA. Results of our present study clearly suggest that female DA may play a prominent role in determining the higher reproductive success of large males.

Disentangling the effects of male mating behavior and female choice in order to assess the importance of DA can be challenging. Indeed, the higher spawning probability and per capita egg production to large males in the present study may have been facilitated by male behavior, not female allocation. Large, dominant males may be more aggressive [52] and active in initiating spawning than small males, and this could have led to higher spawning probability, egg production and fertilization rate [56], [57]. In zebrafish, however, small subordinate males have been shown to sire more offspring compared to larger-sized subordinates in the presence of dominant males, potentially owing to their enhanced manoeuvrability, which enabled them to get closer to females during mating [58]. We cannot exclude the possibility that, in our study, large males received more eggs due to their more aggressive mating behavior compared to the small males. However, because there is no evidence in zebrafish that large males initiate spawnings more frequently than small males [59], we can be relatively confident that we have documented a pattern of DA rather than male ability to stimulate female egg release. Obviously, the documented correlation between female DA and male body size does not prove causality. Any variable that is correlated with male body size might contribute to the patterns observed. For example, male body size might correlate positively with mating experience. Hence, one mechanistic explanation for the higher spawning probability of zebrafish females coupled with large males compared to small males could be related to the greater spawning experience of large males. Also other factors might correlate with male body size, including male's dominance rank [52], and future studies should focus on processes behind the DA pattern revealed in the present study.

Evidently, social factors are important in influencing mate choice, and experience with different male phenotypes affects mate preferences in fish [27], [28], [32], [60]. Therefore, it is reasonable to assume that the mating preference and allocation pattern may be adjusted, at least to certain degree, by changing the social environment. We predicted that low frequency of high quality-males (i.e., females conditioned to small males) would lead to increased sampling costs among females and shifts their preferences so that less attractive males are also accepted [26], [31]. In contrast to our expectations, changes in social environment did not induce plasticity in female propensity to spawn or strongly alter the egg allocation pattern. Instead females consistently had higher spawning probability when coupled with large males independent of the conditioning treatment, although there was a trend for a weakened discrimination against small males in our control group. It has been reported that strong social preferences are formed when zebrafish are juveniles [61], [62], and conditioning mature adults for a relatively short time period, as done in our study, was potentially not enough to create strong social imprinting. More work is needed with longer-term conditioning to verify the robustness of our conclusions.

Although short term changes in adult social environment might not induce a plastic response in zebrafish mate preference and allocation pattern, changes in density and sex-ratio might alter the reproductive success of different-sized males. It has been shown that large males have higher reproductive success, potentially reflecting female DA, in low density (3 individual groups), but not in high density (15 individual groups) [23], [63]. In high density the female oviposition may be interrupted more frequently and the risk of egg cannibalism may be enhanced [23], thus high density may introduce relatively high fitness costs to the female. Consequently, females may reduce their selectivity and allocate equal amounts of reproductive resources to small and large males [51]. Alternatively, this result could have been a consequence of intense male-male competition where male interactions might play a predominant role and overwhelm female preferences. We admit that the results from isolated monogamous studies, such as ours, may not always be accurate indicators of DA in nature. In fact, it is possible that female mate choice and allocation can be altered by the presence of other males and females, i.e., by more realistic social interactions [57]. However, it has been shown that wild zebrafish spawn in pairs rather than in groups [25] and therefore the allocation pattern is potentially less affected by density-dependent interactions among individuals in nature.

We showed that females had higher probability to spawn and they allocated more eggs towards large males compared to small ones. Although large zebrafish males may be preferred by females [24] and be superior (e.g., more dominant) in many ways compared to small ones, large males may also have a selective disadvantage under specific environmental conditions [58], [64], which were not part of our experimental design. As competitive environments fluctuate, there may be no single optimal phenotype, and to maximize fitness, individuals must match their phenotype to the specific competitive challenge they are likely to encounter [64], [65]. Although zebrafish females had higher spawning probability when coupled with the large males compared to small ones, some females spawned with and produced eggs to small males as well. This behavior was particularly pronounced among females held with random-sized males, an environment that reflects the variation in male body size occurring in nature. It is possible that owing to certain size-dependent differences in male reproductive behavior, small males have a selective advantage under specific competitive contexts [58], thus selection for male body size is not expected to be directional. We nevertheless showed in zebrafish that female allocation patterns towards large males was relatively persistent across different social environments, potentially reflecting high fitness benefits females can receive from large males in benign, laboratory environment. A future goal could be to broaden the search for the mechanisms causing female mate preference and to study how these mechanisms interact. It would be important, in particular, to know to what degree environmental variables (e.g., predation risk, competition, social interactions) induce variation in mate preference and egg allocation, how repeatable the results are at the individual level and how the variation in female preferences affects the mean offspring fitness.
